# Effect of pulsed electromagnetic field as an intervention for patients with quadriceps weakness after anterior cruciate ligament reconstruction: a double-blinded, randomized-controlled trial

**DOI:** 10.1186/s13063-022-06674-2

**Published:** 2022-09-12

**Authors:** Michael Tim-Yun Ong, Gene Chi-Wai Man, Lawrence Chun-Man Lau, Xin He, Jihong Qiu, Qianwen Wang, Matthew Chun-Sing Chow, Ben Chi-Yin Choi, Mingqian Yu, Patrick Shu-Hang Yung

**Affiliations:** Department of Orthopaedics and Traumatology, Faculty of Medicine, The Chinese University of Hong Kong, Hong Kong SAR, China

**Keywords:** Pulsed electromagnetic field (PEMF), Quadriceps strength, Anterior cruciate ligament (ACL), Anterior cruciate ligament reconstructions (ACLR)

## Abstract

**Background:**

The ultimate goal of anterior cruciate ligament reconstructions (ACLR) is to fulfil the return-to-play (RTP) criteria. Quadriceps muscle strength is one of the key determinants for a patient’s successful return-to-play after ACLR. Quadriceps muscle atrophy can persist beyond the completion of the rehabilitation program in almost half the patients and the reason behind this is still unknown. There are emerging evidences showing that pulsed electromagnetic field (PEMF) can modulate mitochondrial activities for muscle gain. PEMF exposure on top of regular exercise training may promote muscle regeneration and tissue healing.

**Methods:**

This is a double-blinded, randomized controlled trial to investigate the effects of PEMF treatment during the postoperative period on quadriceps muscle strength in ACL injured patient. Adult patients (aged 18–30) with a unilateral ACL injury, total quadriceps muscle volume is equal or more than 7% deficit on involved leg compared with uninvolved leg, sporting injury with a Tegner score of 7+, and both knees without a history of injury/prior surgery will be recruited. To estimate the improvement of patients, isokinetic muscle assessment, ultrasound imaging and MRI for quadriceps muscle thickness, self-reported outcomes with questionnaires, KT-1000 for knee laxity and biomechanical analysis, and Xtreme CT for bone mineral density will be performed. To investigate the mechanism of PEMF therapy on increasing quadriceps strength, samples of blood serum will be drawn before and after intervention.

**Discussion:**

This is the first trial evaluating the effects of PEMF on quadriceps muscle recovery after ACLR. The proposed study addresses a huge research gap by evaluating practical use of PEMF as part of rehabilitation. The proposed study will provide much needed scientific support in the use of this noninvasive treatment modality to facilitate recovery of quadriceps strength after PEMF.

**Trial registration:**

ClinicalTrials.gov NCT05184023. Registered on 5 January 2022

**Supplementary Information:**

The online version contains supplementary material available at 10.1186/s13063-022-06674-2.

## Administrative information

Note: the numbers in curly brackets in this protocol refer to SPIRIT checklist item numbers. The order of the items has been modified to group similar items (see http://www.equator-network.org/reporting-guidelines/spirit-2013-statement-defining-standard-protocol-items-for-clinical-trials/).

**Table:** 

**Title {1}**	**A Double-Blinded, Randomized-Controlled-Trial to Investigate the Effect of Pulsed Electromagnetic Field (PEMF) for Patients with Quadriceps Weakness After Anterior Cruciate Ligament Reconstruction (ACLR)**
Trial registration {2a and 2b}.	Clinical.Trials.gov identifier: NCT05184023, 5th January 2022
Protocol version {3}	Original version dated: 14.5.2021 First revision dated: 16.12.2021 • Primary reason for revision: Amendment suggested by the ethics committee for the further justification of the required sample size
Funding {4}	Fully funded by the Health and Medical Research Fund (HMRF), Food and Health Bureau of the Government of the Hong Kong Special Administrative Region (Ref: 19202461). The funder has no role in the design of the study and collection, analysis, interpretation of data, and in writing the manuscript.
Author details {5a}	Michael Tim-Yun Ong^1^, Gene Chi-Wai Man^1^, Lawrence Chun-Man Lau^1^, Xin He^1^, Jihong Qiu^1^, Qianwen Wang^1^, Matthew Chun-Sing Chow^1^, Ben Choi^1^, Mingqian Yu^1^, Patrick Shu-Hang Yung^1^ ^1^ Department of Orthopaedics and Traumatology, Faculty of Medicine, The Chinese University of Hong Kong, Hong Kong SAR, China
Name and contact information for the trial sponsor {5b}	The Chinese University of Hong Kong Department of Orthopaedics and Traumatology Dr. Michael Tim Yun Ong Rm 74034, 5/F, Lui Che Woo Clinical Science Building, Prince of Wales Hospital, Shatin, Hong Kong SAR, China michael.ong@cuhk.edu.hk
Role of sponsor {5c}	Role of study sponsor: role and ultimate authority in study design; collection, management, analysis, and interpretation of the data; writing of the report; and the decision to submit the report for publication. Role of study funder: no roles in the collection, management, analysis, and interpretation of the data; writing of the report; or the decision to submit the report for publication.

## Background and rationale {6a}

In Hong Kong, over 3000 anterior cruciate ligament reconstructions (ACLR) are performed each year in order to restore knee function after an ACL injury. The ultimate goal of ACLR is to fulfil the return-to-play (RTP) criteria. Despite successful surgery and a demanding rehabilitation process, some athletes still fail to comply with RTP. And for those who achieve RTP, 23% of those who return to their sports would suffer a second ACL injury [1].

After ACLR, the patient would undergo post-operative rehabilitation. The rehabilitation gradually helps patients to regain mobility and strength, where most of the patients would be expected to RTP by 12 months. Despite comprehensive rehabilitation programs, a systematic review showed up to 35% of patients would fail to return to their preinjury level of play [2]. Additionally, these patients were found to be 6 times more likely to sustain a second ACL injury within 24 months after ACLR than the healthy population [3].

Quadriceps muscle strength is one of the key determinants for a patient’s successful return-to-play after ACLR, by which muscle size is a crucial factor of muscle strength. Quadriceps muscle atrophy is unavoidable after ACLR, but rehabilitation aids in recovering quadriceps muscle mass. However, despite good compliance, some patients respond poorly and fail to regain muscle mass. Quadriceps muscle atrophy can persist beyond the completion of the rehabilitation program in almost half the patients and the reason behind this is still unknown. This represents an area requiring significant investigation, as quadriceps muscle atrophy and weakness have been shown to be predictive of poor knee function, decreased performance in sports, and increased risk of reinjury. There are emerging evidences showing that pulsed electromagnetic field (PEMF) can modulate mitochondrial activities for muscle gain. PEMF exposure on top of regular exercise training may promote muscle regeneration and tissue healing.

Exercise-induced increase in muscle mass relies heavily on mechanical and metabolic adaptations, which depend on mechanically gated calcium channels, especially transient receptor potential canonical 1 (TRPC1). Exercise promotes muscle development, while physical inactivity could result in muscle atrophy. TRPC1 was implicated in load-dependent oxidative pathways related to muscle development, and the expression of TRPC1 diminished in disuse [4]. Concurrently, mitochondrial adaptations also play a fundamental role in muscular and systemic metabolic homeostasis which are largely dependent on the transcriptional coactivator, peroxisome proliferator-activated receptor-gamma coactivator-1α (PGC-1α) [4]. PGC-1α is a key determinant factor in muscle development and maintenance due to its role to mediate expression of myokines, such as irisin, brain-derived neurotrophic factor (BDNF), interleukin 6 (IL-6), and myostatin, which are all related to the regulation of myogenesis. Therefore, mediating transient receptor potential canonical 1 (TRPC-1) and PGC-1α could be a possible way to promote myogenesis.

Pulsed electromagnetic field (PEMF) treatment has been shown to modulate mitochondrial activities for muscle gain. Molecular processes are sensitive to the magnetic flux at extremely low frequency (<300 Hz), which are equivalent to the Earth’s natural magnetic resonance (Schumann resonance). Applying magnetic fields at these low frequencies in the form of magnetic pulses, like a timely push to swing. The pulsing action will enhance the human magnetosensitive processes, which include the electron transport chain in mitochondria, calmodulin signalling, and ion channels. The affected signal transduction pathways further influence the downstream myogenesis [5]. Therefore, a pulsed electromagnetic field (PEMF) may be a possible intervention to amplify molecular processes in favour of myogenesis [6]. PEMF therapy has been demonstrated to enhance cellular activity related to tissue healing [7], and offer beneficial effects such as pain relief, anti-inflammation, and oedema resolution [8]. Numerous variables in the delivery of PEMF signals (i.e. magnetic flux, frequencies, signal curve, polarity, etc.) may have specific biological implications. Our group (Prof. Alfredo Franco-Obregon) showed that brief 10-min exposure of 1.5mT amplitude of PEMF (Quantum Tx) on myoblast in vitro could activate myogenesis. It is done so by stimulating the TRPC-1-mediated calcium entry and downstream factors as well as PGC-1α; this mechanism (mentioned above) is similar to the myogenesis in exercise [4] (Additional file 1). PGC-1α gene expression is influenced by every major signalling pathway that is activated in a contracting muscle [9]. Additionally, it plays an important role in regulating the production and secretion of myokine. Therefore, PEMF exposure on top of regular exercise training may promote the secretion of myokine and in turn promote muscle regeneration.

PEMF treatment utilizes low energy magnetic field to stimulate mitochondrial activity in skeletal muscle. It leads to a boost in repairing and regenerating muscle via the release of myokines. These findings laid grounds to implement PEMF treatment for post-surgical patients to enhance muscle regeneration in periods where physical activity is not possible. Also, it helps to set up the treatment regime for the PEMF device to impose myogenic effects, including exposure time per session, the number of treatment sessions and the duration of treatment. In our previous work submitted for peer-review, a positive trend was shown for the gain in muscle strength and size in the early postoperative period after ACLR. To our best knowledge, this proposed study aims to apply PEMF as an intervention for patients with myokine-mediated persistent quadriceps muscle atrophy after ACLR. In turn, it would be the first study to examine the correlation between PEMF and postoperative quadriceps muscle endurance, strength, and mass. This study aims to conduct a double-blinded, randomized controlled trial to investigate the effects of PEMF treatment during the late postoperative period on quadriceps muscle strength in ACL injured patient. Muscle endurance could only be investigated in late postoperative period. The investigators hypothesize that PEMF treatment is effective to reduce muscle weakness and promote gain in quadriceps muscle strength in ACLR patients. Importantly, this study may shed light on the effectiveness of this non-invasive, safe biophysical intervention to the general population with persistent muscle atrophy or muscle wasting to potentially improve their well-being and health.

### Objectives {7}

This study aims to conduct a double-blinded, randomized controlled trial to investigate the effects of PEMF treatment on quadriceps muscle endurance and strength in patients after ACLR.

### Trial design {8}

This study is a randomized, double-blinded, placebo-controlled clinical trial to investigate the effects of pulsed electromagnetic field (PEMF) for patients with quadriceps muscle weakness after anterior cruciate ligament reconstruction (ACLR). The intervention groups receives PEMF treatment whereas the control group receives the treatment-as-usual (TAU). Both treatments are conducted in clinical practices that are randomly allocated to either the intervention or the control condition. Participants are therefore randomized as their allocation depends on their practice being an intervention or a control practice.

Data is assessed at the four measurement time points from the participants:

- Baseline/inclusion
- 1st follow-up: post-op at 5 months
- 2nd follow-up: post-op at 6 months
- 3rd follow-up: post-op at 12 months

## Methods: participants, interventions, and outcomes

### Study setting {9}

Patients are recruited from a local hospital in Hong Kong. These patients will be followed up at the Orthopaedics Outpatient Clinic at the Prince of Wales Hospital for clinical examination and questionnaire filling. While for the muscle assessments and muscle imaging, it will be conducted at the Physiotherapy Department and Department of Imaging and Interventional Radiology at the Prince of Wales Hospital, respectively.

### Eligibility criteria {10}

The inclusion criteria are as follows:

1. Aged 18–30 with a unilateral ACL injury
2. Sporting injury with a Tegner score of 7+
3. LSI for quadriceps strength <70% of the contralateral leg at 4 months post-op
4. Both knees without a history of injury/prior surgery

The exclusion criteria are as follows:

1. Ages smaller than 18 years old or greater than 30 years old
2. Any concomitant bone fracture, major meniscus injury or full-thickness chondral injuries requiring altered rehabilitation program post-op
3. Preoperative radiographic signs of arthritis
4. Metal implants that would cause interference on MRI
5. Non-HS graft for ACLR
6. Patient non-compliance to the rehabilitation program
7. Pregnancy or possibility of pregnancy

### Who will take informed consent? {26a}

Trained research assistants will obtain written informed consent from all participants prior to their participation of this study. Our research assistants will first explain to eligible participants our programme in detail.

### Additional consent provisions for collection and use of participant data and biological specimens {26b}

Consent of participant data and biological specimens are also included in the informed consent. Blood specimens in this study will be disposed after testing and will not be used for genetic analysis or used in other studies. Every participant will be represented by an ID number and personal information such as name, address, and telephone number will be kept confidential before, during, and after the study. The trial will be conducted in accordance with the Declaration of Helsinki.

### Interventions

#### Explanation for the choice of comparators {6b}

Patients randomized to the control arm will received a sham exposure with the same PEMF device. As the active-pulse electromagnetic field device does not produce heat or cause any sensation to the tissue, this can ensure the participants to be blinded to the treatment.

#### Intervention description {11a}

Participants in the intervention group will be exposed to PEMF treatment by a PEMF device (Quantum Tx, Singapore). The active pulse electromagnetic field device does not produce heat or cause any sensation to the tissue which allows the participants to be blinded to the treatment. In brief, alternate legs will be exposed to PEMF or sham treatment for 10 min per session. The treatment regime will run twice a week for 8 weeks, summing up 16 sessions of PEMF or sham exposure in total. According to our pilot study in Singapore, such alternating leg exposure establishes a cross-feeding scenario whereby muscle recovery is fully supported and systemic mitochondrial and myokine release are optimized.

The procedure of PEMF treatment is shown as follows:

- The subject will be seated at a 90° position on a chair.
- The solenoids of the PEMF device will be adjusted to be over the thigh (quadriceps and hamstring).
- The options of the appliance will be adjusted to 1 mT, 15Hz on one leg for 10 min.

#### Criteria for discontinuing or modifying allocated interventions {11b}

The PEMF treatment has been declared safe to users based on previous studies and FDA approvals on certain applications [10]. Adverse effects during PEMF therapy will be closely monitored by the designated research staff. In the case of a patient experiencing intolerable discomfort or pain, PEMF treatment will only be delivered on the contralateral side until the aggravated local inflammatory response due to PEMF-driven mitochondrial activation subsides (determined by medical staff). As the treatment is performed at the site of PWH, medical staffs are readily available for the assessment and management of any potential serious adverse effects. In the event of a serious adverse reaction to the PEMF treatment, medical staff will perform the evaluation determining whether the patient would need to be immediately removed from the study in their best interest to safeguard their health.

#### Strategies to improve adherence to interventions {11c}

Patients will be contacted 1 week before the treatment session or assessment to enhance the attendance rate. Special rehabilitation session or assessment session on the weekend or in the evening will be arranged under special circumstances to enhance subject compliance. Patients who have missed the scheduled session will be contacted by telephone within 1 week for a makeup session. In the case of patient non-compliance, study personnel would remove the patient from the study only when multiple failed attempts to contact and convince the patient occur. If the patient chooses to withdraw from the study before the end of the study period, the reason and termination date will be recorded. As far as possible, we will invite patients who would like to withdraw to attend the final assessment.

#### Relevant concomitant care permitted or prohibited during the trial {11d}

Participants remain on their standard treatment and medication procedures throughout the study period, and clinicians are advised to manage participants in the usual manner subject to the caveats outlined above.

#### Provisions for post-trial care {30}

Not applicable, since ancillary and post-trial care is provided within the standard care.

### Outcomes {12}

The primary outcome measures are the change of peak torque of isokinetic muscle strength and the change of fatigue index (FI) of isokinetic muscle strength with or without PEMF during 8 months of study follow-up. The peak torque, measured in Newton metre (Nm), will be the single highest repetition value within the 30 repetitions in the isokinetic muscle strength test, whereas the FI will be used to calculate the percent decrease for each variable which reflected the muscle endurance. Secondary outcome measures include (1) assessment of quadriceps muscle volume and muscle thickness over 8 months of follow-up expressed as changes in cross-sectional area (cm^2^) with baseline, (2) evaluation of serum myokines using a human myokine magnetic bead panel (Milipore) over 8 months of follow-up, (3) measurement of passive knee laxity using KT-1000 knee ligament arthrometer (MEDmetric Corp, San Diego, CA, USA) over 8 months of follow-up, (4) analysis on the reaming size of the bone using XtremeCt II over 8 months of follow-up and expressed as mm, (5) evaluation of ground reaction force will be evaluated by a synchronized force plate at the centre of the capture volume at 1000 Hz over 8 months of follow-up, (6) assessment of knee joint moments will be assessed by the skin marker-based motion analysis system over 8 months of follow-up, (7) distance by single leg hop test during 8 months of follow-up will assessed by single leg hop distance (in cm), and (8) patient-reported outcome measures for assessing pain, disability, and activity level on knee function will be evaluated during the 18 months of follow-up.

### Participant timeline {13}

**Fig. 1:**
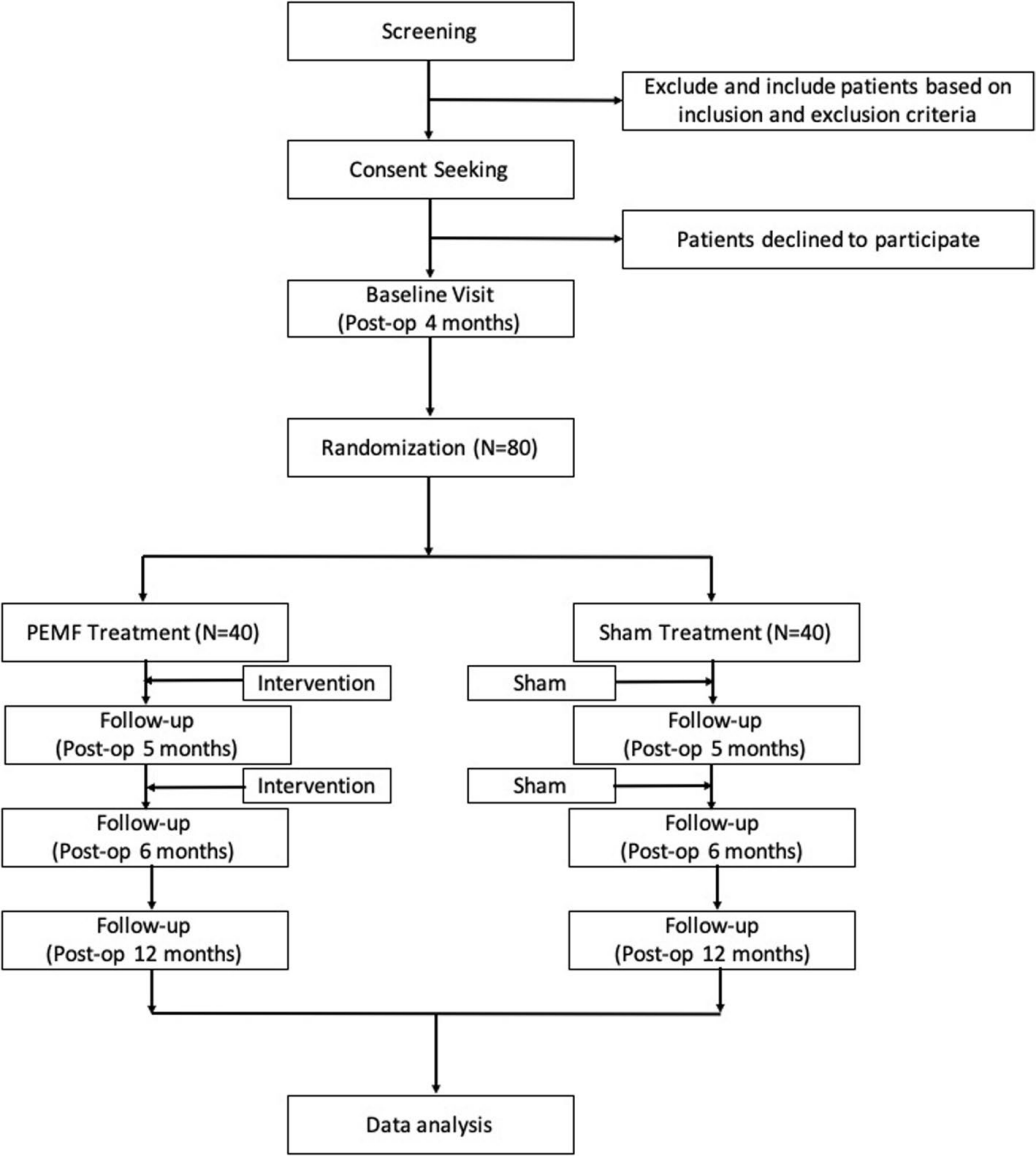
Study flowchart

The study flowchart is illustrated in Fig. 1.

### Sample size {14}

Quadriceps muscle strength will be employed as the primary outcome for sample size estimation. Based on our pilot work, we detected a 30 Nm (SD 60 Nm) mean difference between atrophy and non-atrophy group. A sample size of 30 in each group will have 90% power to detect a significant difference using a two-sided independent t test with a 0.05 significance level (PASS 11.0; NCSS Statistical Software, LLC, East Kaysville, UT, USA). Taking account of 25% dropout rate, we further increase the sample size to *n* = 40 for each arm (total *n* = 80).

### Recruitment {15}

Patients with persisting quadriceps muscle weakness after ACLR will be consecutively recruited. These patients will be actively screened and recruited from the Department of Orthopaedics and Traumatology, Prince of Wales Hospital, Hong Kong, based on inclusion and exclusion criteria. Centre staff will help identify eligible individuals and refer to us for screening. The principal investigator will then explain the study procedures to the patients. Patients who agree to participate in the study will come back on a scheduled visit for baseline investigation. The completion of the trial is expected to take 36 months.

## Assignment of interventions: allocation

### Sequence generation {16a}

#### Randomization and blinding

A total of 80 patients will be enrolled. Participants will be randomized into 1:1 allocation, blocked randomization with 40 participants in the PEMF group and 40 participants in the sham group. Each allocation will be assigned with a unique radio frequency identification (RFID) (generated during block randomization by the PEMF supplier service) recognizable by the PEMF machine. Each card can be shared among 10 subjects, and the subjects will be assigned an RFID by which the PEMF or sham treatment will be randomly assigned to the RFID. A biostatistician who does not participate in the recruitment of patients will oversee the randomization. Hence, both participants and the research personnel are blinded and participants will use the RFID to complete the assigned treatment without knowing which treatment they are receiving.

### Concealment mechanism {16b}

All randomization will be done using a computer randomization programme before the intervention. This will be oversees by a biostatistician who does not involved in the recruitment of patients and data analysis. The allocation concealment will be ensured, as the statistician will not release the randomization code until the baseline examination has been completed. Patients and investigators are fully blinded until the completion of treatment. Outcome assessors and statistician are also blinded.

### Implementation {16c}

The principal investigator will enrol participants. The computer randomization programme will generate the allocation sequence at a 1:1 ratio. An independent research staff will assign participants to interventions.

## Assignment of interventions: blinding

### Who will be blinded {17a}

Participants are assumed to be not blinded to the intervention. Nevertheless, they will be instructed not to discuss their allocation with other participants or outcome assessors. To reduce the possible expectancy bias, participants will be explained at the time of giving consent that there is no evidence to support a difference in cognitive benefit between the two arms. The research assistant who assists in consent seeking and monitoring of progress and adverse events will not be involved in outcome assessment. The assessor who will be another trained research assistant will be blinded to the randomization status and will not be involved in the intervention. The statistician responsible for the randomization is not involved in other parts of the study including data analysis.

### Procedure for unblinding if needed {17b}

In normal circumstances, the blinding will be maintained unless a serious adverse event occurs. Unblinded participants will then exit the trial and the medical conditions will be managed accordingly. The management results will be recorded on the clinical report form and reported to the Joint Clinical Research Ethics Committee of the Chinese University of Hong Kong and the New Territories East Cluster of the Hospital Authority.

## Data collection and management

### Plans for assessment and collection of outcomes {18a}

#### Anthropometric measurement

Body mass index (BMI) would be measured with height and weight and calculated at post-op 4, 5, 6, and 12 months. We would measure the waist circumference, and the subcutaneous fat would be measured using skinfold techniques at the triceps brachii and biceps brachii.

#### Isokinetic muscle assessment for maximal strength and endurance

The dynamometer (Biodex System 4, Biodex Medical Systems Inc., New York, USA) will be used. Subjects will perform a standardized warm-up exercise (5-min cycling) followed by the fatigue test. Five repetitions at speed of 180°/s will be done first to familiarize the patient. Concentric/concentric contractions of knee extension/flexion will be tested at 180°/s for 30 repetitions. Subjects will be seated on the dynamometer chair with the hip flexed to 85°. The re-test reliability has been proven for this protocol [11]. The relatively fast speed is chosen to perform the fatigue test since the fast-twitch muscle fibres are expected to fatigue faster at high speeds [12]. Peak torque will be the single highest repetition value within the 30 repetitions [13]. Muscle endurance could be reflected by fatigue test, where the trends for peak torque, work, and power will be examined. The FI will be used to calculate the percent decrease for each variable [13]. Percent decrease = 100 − [(last 5 repetitions/first 5 repetitions) × 100]. For individuals that did not achieve their peak torque within the first 3 repetitions, a second F.T. will be calculated as follows. Percent decrease = [100 − [(last 5 repetitions/highest consecutive 5 repetitions) × 100], where the highest consecutive five repetitions will be determined by values attained from the repetitions immediately before and following, the single highest repetition value [13].

#### Muscle volume and quality assessment

Muscle volumes of quadriceps muscle will be measured using a 1.5- or 3.0-Tesla MRI Scanner. Axial (3-mm thick cut) T1W images will be obtained from the anterior superior iliac spine (ASIS) to the patella. Quadriceps muscles will be manually outlined in each axial slice. Muscle volume will then be calculated by summing all of the slice-multiplied by slice thickness. The quality of the muscle will be assessed by analysing the fat content of the muscle mass using a technique that has been reported by Reeder et al. [14]. Scans will be performed on both legs before the start of the PEMF treatment (4 months post-op) and only on the injured side repeated after the completion of the 8 weeks PEMF treatment (6 months post-op). The uninjured side will be used as a reference for ‘normal volume’.

In addition, the Aixplorer® ultrasound system and a linear transducer probe with a bandwidth of 2–10 MHz will be used to measure the muscle thickness of vastus medialis (VM), vastus lateralis (VL), and rectus femoris (RF) on both the injured and uninjured leg. Participants will lay supine on a treatment table for the assessment. A measuring tape will be used to locate VM, VL, RF, and the patella by palpation, consequently marked with a pen for reference. RF will be marked at 1/2 of the distance from the ASIS to the superior pole of the patella, VM will be located at 1/5 of the distance away from the midpoint of the medial patella border to the ASIS, and VL will be noted at 1/3 of the distance from the midpoint of the lateral patella border to the ASIS. After locating the anatomical points, excess contact gel was applied on these points. The transducer probe will be aligned in the transverse plane and moved along the entire muscle bundle to capture a view of the VM, VL, and RF.

#### Serum myokine evaluation

Phlebotomy (5ml) will be performed on the day before PEMF treatment, at 4 and 8 weeks after commencement of treatment, and at 8 months after the commencement of intervention. The serum will be prepared by centrifugation and kept in a −80° freezer until use. The serum will be prepared by centrifugation and kept in a −80° freezer until use. Quantitative analysis for myokines and proteins related to muscle metabolism will be performed by Human Myokine Magnetic Bead Panel (Millipore) with Bioplex-200 bead-based suspension assay system, or enzyme-linked immunosorbent assay (ELISA). These myokines include brain-derived neurotrophic factor (BDNF), fibroblast growth factor-21 (FGF-21), interleukin-6 (IL-6), IL-15, irisin, myostatin (MSTN)/GDF8, insulin-like growth factor 1 (IGF-1), FGF-2, IL-8, follistatin, musclin, myonectin, decorin, meteorin-like, osteopontin, secreted protein acidic and rich in cysteine (SPARC), klotho, procollagen type III N-terminal peptide (P3NP), and C-terminal of troponin T1 (TNNT1).

#### Passive knee laxity

To measure anterior-posterior knee laxity, the KT-1000 knee ligament arthrometer (MEDmetric Corp, San Diego, CA, USA) will be used. A manual force test will be applied until a 30lb sound signal is activated. Three trials will be performed. A side difference of 3 mm above is considered clinically relevant [15].

#### Reaming size of the bone

We assessed the reaming size of the bone (in mm) on the involved side of the distal tibia using high resolution-peripheral quantitative computed tomography (HR-pQCT) (XtremeCT II; Scanco Medical AG, Brüttisellen, Switzerland). This can provide an analysis of bone microarchitecture at human peripheral sites with high spatial resolution and low exposure to radiation. Bone mineral density (BMD, calibrated with hydroxyapatite standards) and finite element analysis of subchondral bone over the knee joint will be performed using the Scanco software. Scans will be performed on both legs before the start of the PEMF treatment (4 months post-op), and on the injured side after the completion of the PEMF treatment (6 and 12 months post-op). The uninjured side will be used as a reference for ‘normal’ bone condition.

#### Biomechanical analysis

##### Ground reaction force and knee joint moments

The kinematics will be assessed by the skin marker-based motion analysis system (Vicon MX, Oxford, UK) with the lower-body marker setup followed the OSTRC standard protocol using 16-camera and 16 reflective skin markers. The kinetic variables including vertical and horizontal ground reaction force (GRF) and joint moments will be evaluated by a synchronized force plate (0.60 × 0.40 m, model OR6-7, AMTI, Watertown, MA) at the centre of the capture volume at 1000 Hz. From the single leg hop (SLH) task, the knee kinematics in the landing phase is the key measurements including knee flexion angle at initial contact and the range of knee motions (flexion-extension, valgus-varus, external-internal rotation). The hip and ankle flexion angle at initial contact will also be recorded. The GRF will be normalized to body weight and moments will be normalized for body weight ×limb length to make the comparison between subjects possible. All analyses for moments and angles were performed in the anatomical sagittal (XY) plane and are counted as positive for flexion angles and extension moments. The start of the rise in the vertical GRF will be used to determine initial ground contact. From the SLS task, the knee kinematics is the key measurements including the range of motions (flexion-extension, valgus-varus, external-internal rotation) and the frequency of the relative tibiofemoral angular displacements from the kinematics curve, which will be quantified by the number of peak appearance. The kinematic and kinetic variables were imported and calculated in the Matlab (The Mathworks Inc., Natick, Massachusetts, USA). A biomechanical analysis will be performed on all patients. At the same time, the motion will be videotaped by high-speed cameras.

##### Single-leg hop distance

The SLH test will be performed as previous studies reported [16]. The test reflects the knee stability during single-leg hop landing, which is directly influenced by quadriceps strength. Three trials will be performed with each leg followed by familiarization. The SLH will be deemed valid if the patient can achieve maximal hop distance while maintaining balance for at least 2s after landing.

##### Patient-reported outcome measures

To assess overall health and knee-related symptoms, pain, functionality, and everyday ability and activity level, patients will be asked to complete the following questionnaires: Visual Analogue Scale (VAS) [17], Tegner score [18], International Knee Documentation Committee score (IKDC) [19], Lysholm Score [20], and International Physical Activity Questionnaire [21]. These clinical scores will be calculated using their corresponding analysis tools.

The assessment schedule is shown in Table 1.

**Table 1:** Assessment schedule

Assessments	Baseline (post-op 4 months)	Follow-up (post-op 5 months)	Follow-up (post-op 6 months)	Follow-up (post-op 12 months)
Anthropometric measurement	✓	✓	✓	✓
Isokinetic assessment	✓	✓	✓	✓
MRI muscle thickness assessment	✓		✓	
Ultrasound imaging muscle thickness	✓	✓	✓	✓
Serum myokine evaluation	✓	✓	✓	✓
Passive Knee Laxity by KT-1000	✓	✓	✓	✓
Reaming size of the bone by XtremeCT	✓	✓	✓	✓
Ground reaction force	✓	✓	✓	✓
Knee Joint moments	✓	✓	✓	✓
Single leg hop distance		✓	✓	✓
Visual Analogue Scale	✓	✓		✓
Tegner Activity Score	✓	✓	✓	✓
International Knee Documentation Committee (IKDC) Questionnaire	✓	✓	✓	✓
Lysholm score	✓	✓	✓	✓
The International Physical Activity Questionnaires (IPAQ)	✓	✓	✓	✓
Adverse events		✓	✓	✓

### Plans to promote participant retention and complete follow-up {18b}

Patients will be contacted 1 week before the treatment session or assessment to enhance the attendance rate. Special rehabilitation session or assessment session on the weekend or in the evening will be arranged under special circumstances to enhance subject compliance. Patients who default a scheduled appointment will be contacted by the investigators to re-arrange another appointment within 1 week.

### Data management {19}

Clinical data will be collected and recorded by trained research assistants in our research centre. Clinical examination data will be entered on case report forms and then entered electronically. Consistency checks by another technician will be performed to ensure data entry accuracy. All data will be stored in password-protected computers. The study will be conducted in compliance with Good Clinical Practices to ensure the rights and well-being of the participants and that the data collected are complete and verifiable from source documents. Patients are free to withdraw from the study at any time without giving any reasons, and their medical care or legal rights will not be affected. Patient files will be maintained in storage for a period of 3 years after completion of the study.

### Confidentiality {27}

All personal information and consents on enrolled patients collected on paper versions will be kept in locked units at the participating practice and later at the coordinating centre to be archived. Each patient will be assigned an identification code. All information collected and inputted on the electronic database will be based on the identification code and therefore does not contain any personalized information that enables the identification of the patient. The document containing the information of the identification code and the identity of the patient will be kept separate from the study data files and data sheets. The patient identification code list and database can only be retrieved by dedicated study team members or be inspected by study monitors for quality checking and verification.

### Plans for collection, laboratory evaluation and storage of biological specimens for genetic or molecular analysis in this trial/future use {33}

Blood will be collected at the clinical sites for future diagnostic test development and evaluation at treatment sites. After arrival at the local research laboratory at each site, the samples will be collected, transported, stored, and prepared according to local protocols. Blood will be stored at 2–8 °C before handling within the required time. All samples collected during the trial will be labelled with the patients’ identification code and will not contain any identifiable data. Patients have the option of consenting to their samples being stored for future research uses. Samples will be stored anonymously at a central location for a minimum of 5 years and a maximum of 10 years after completion of the study, after which these specimens will be destroyed by incineration according to local guidelines and protocols. The potential usage of the stored samples is included in the informed consent form. Nevertheless, further usage of the samples will need to be approved by the institutional ethical committee.

## Statistical methods

### Statistical methods for primary and secondary outcomes {20a}

Statistical analysis will be performed using SPSS software (SPSS 26.0). The normality of the data will be tested using the Kolmogorov-Smirnov test. A repeated-measures one-way analysis of variance (ANOVA) will be used to compare muscle volume (MRI), quadriceps muscle strength (isokinetic assessment), serum myokine levels, and results of questionnaires at the aforementioned time points. A non-parametric Mann-Whitney *U* test will be used to compare questionnaire results (ordinal data) between PEMF and Sham groups.

### Interim analyses {21b}

Interim analysis will be performed when approximately 10% of our sample have completed follow-up assessments. The preliminary findings will be presented in conference to promote our study.

### Methods for additional analyses (e.g. subgroup analyses) {20b}

Additional analyses will include within-trial and long-term cost-effectiveness analyses. Analyses will report differences in cost of service use between groups using quality-adjusted life-years (QALYs) derived from EQ-5D-5L. The primary within-trial analysis will compare direct costs and 12-month outcomes of participants randomized to the intervention group (PEMF treatment) and the control group (sham treatment). Secondary analyses will adopt a societal perspective taking account of productivity costs and out-of-pocket expenditures incurred by participants. Data collected at baseline, 6 months, and 12 months will be utilized to estimate incremental cost-effectiveness ratios comparing the intervention with the control group. Quality of life (derived from the EQ-5D-5L) over the study period will be used to generate QALYs. Sensitivity analyses will consider key cost drivers and factors that might affect the outcomes measured to explore uncertainty in the conclusions drawn. Utility values derived from the SF36 (SF6D) will be included as a sensitivity analysis

### Methods in analysis to handle protocol non-adherence and any statistical methods to handle missing data {20c}

Data will be analysed on an intention-to-treat basis to include all participants based on the random allocation regardless of whether the intervention has been received or not. Adherence to intervention or withdrawal, which is recorded by our trained research assistants on a weekly basis, serves as a covariate and will be included in the data analysis. Missing data will be checked, and where appropriate, multiple imputations will be used and sensitivity analyses will be conducted.

### Plans to give access to the full protocol, participant-level data, and statistical code {31c}

The protocol has been uploaded on ClinicalTrials.gov (ID: NCT05184023). The data of this study will also be available from the principal investigator upon reasonable request.

## Oversight and monitoring

### Composition of the coordinating centre and trial steering committee {5d}

The principal investigator is responsible for the design of the study and the coordination of different cooperation partners. The research team comprises the trial steering committee responsible for the recruitment, assessments, output delivery, and data analysis.

### Composition of the data monitoring committee, its role and reporting structure {21a}

The principal investigator and co-investigators will monitor the data collection and storage to ensure that the data is kept and used in accordance with the protocol. A statistician, who is independent from the sponsor and any competing interests, will be responsible to inspect clinical data collected during the study period, review interim analysis, and report back to the investigators for any action required. Data monitoring committee is not considered as PEMF is a low-risk intervention.

### Adverse event reporting and harms {22}

The PEMF treatment has been declared safe to users based on previous studies and FDA approvals on certain applications [10]. Adverse events will be checked by the investigators at every follow-up visit. Any adverse event, whether they are related to the study or not, will be recorded in the adverse effect report form provided by the Hong Kong Hospital Authority using standard adverse event language. A serious adverse event will be reported to the Hong Kong Hospital Authority Research Ethics Committee within 24 h of the event. The principal investigator will be responsible to follow the management of the serious adverse event until resolution or conclusion. The investigators and the trial steering committee will determine whether an adverse event or serious adverse event is related to the study intervention. The intervention will be stopped immediately if the reported event is considered to be related, with referral to seek medical attention provided by the principal investigator.

### Frequency and plans for auditing trial conduct {23}

As per the university’s requirement, an independent auditor will conduct the annual review throughout the project period.

### Plans for communicating important protocol amendments to relevant parties (e.g. trial participants, ethical committees) {25}

There is no plan for modifying the protocol at this juncture. However, any amendment to the protocol will be submitted by the principal investigator to be approved by the research grant committee of the Health and Medical Research Fund and the ethical committee before implementation. In addition, the trial participants will also be notified as well.

### Dissemination plans {31a}

The research findings will be published in peer-reviewed journals and disseminate to healthcare professionals, the public, and other relevant groups as soon as results are available. The funder has no role or restriction in the decision of publication.

## Discussion

Injury of the ACL mostly concern young and active people who get injured during recreational or professional physical activity. Comprehensive physiotherapy covering both kinesitherapy and physical therapy is aimed at restoring motor skills in patients after ACLR. However, this mobility depends on the adequate level of knee muscles strength, range of motion, and proprioception. Immediately after the reconstruction, the range of motion in the knee joint is often limited by pain and swelling. Such a limitation does not have a positive influence on muscle mass and muscle strength. Patients who failed to regain muscle mass and strength despite rehabilitation could have devastating implications, which includes the disability to perform their preinjury level of sports [22, 23]. Hence, our study proposed the use of PEMF to promote muscle gains in patients that are suffering from persistent muscle atrophy after ACLR. The benefits of PEMF therapy has been demonstrated to enhance cellular activity related to tissue healing [7], and offer beneficial effects such as pain relief, anti-inflammation, and oedema resolution [8]. Additionally, it plays an important role in regulating the production and secretion of myokine. Therefore, PEMF exposure on top of regular exercise training may promote the secretion of myokine and in turn promote muscle regeneration.

The current experimental protocol outlined in this paper can also benefit these patients in many ways. Such that, in the short term, this study would benefit patients who suffered from persistent quadriceps weakness and atrophy after ACLR, thus improving the postoperative outcome. This would potentially lead to a better RTP rate as well as reducing the risk of reinjury, which can be a burden to the health system and society due to the additional treatments and extended time off work. In the medium term, the PEMF treatment can be considered for other musculoskeletal conditions that result in persistent muscle atrophy. In addition, further research examining the role of low field magnetic stimulation in other musculoskeletal effects can further improve the overall outcome of ACLR. This includes enhancing the bone tendon healing of the ACL graft, as well as the potential reduction of post-operative inflammation, subsequently leading to less arthrogenic muscle inhibition (AMI) and therefore, potentially less muscle atrophy in the early post-op period after ACLR. In the long term, the potential use of PEMF can be considered for athletes to improve their muscle functions leading to elevated performance levels.

In the available literature, there is, however, lack of reports on the effectiveness of the use of the PEMF in patients after ACLR. This makes our own protocol a significant contribution to the assessment of the effectiveness of PEMF in the treatment of patients after ACLR. Regardless of our findings, the results will be informative to local patients who failed to regain muscle mass and strength despite rehabilitation and medical clinicians for the potential use of PEMF as a treatment for persistent muscle atrophy.

## Trial status

The protocol version 1 was dated 14 May 2021, and version 2 was dated 16 December 2021. Reasons for revision are described in {3}.

Study recruitment started on 1 September 2021 and the recruitment is still ongoing. The completion of the trial remains scheduled on 1 September 2024.

## Supplementary Information

**Additional file 1.** Informed consent material.

**Additional file 2.** Institutional ethical approval.

**Additional file 3.** Proof of funding.

**Additional file 4.** Research protocol (dated 11 May 2021).

## Data Availability

Any data required to support the protocol can be supplied on request.

## Abbreviations

ACL: Anterior cruciate ligament
ACLR: Anterior cruciate ligament reconstruction
AMI: Arthrogenic muscle inhibition
ANOVA: Analysis of variance
ASIS: Anterior superior iliac spine
BDNF: Brain-derived neurotrophic factor
BMI: Body mass index
ELISA: Enzyme-linked immunosorbent assay
FDA: US Food and Drug Administration
FI: Fatigue Index
FGF-2: Fibroblast growth factor-2
FGF-21: Fibroblast growth factor-21
GRF: Ground reaction force
IGF-1: Insulin-like growth factor-1
IKDC: International Knee Documentation Committee score
IL-6: Interleukin-6
IL-8: Interleukin-8
IL-15: Interleukin-15
HR-pQCT: High resolution-peripheral quantitative computed tomography
MRI: Magnetic resonance imaging
MSTN: Myostatin
Nm: Newton metre
P3NP: Procollagen type III N-terminal peptide
PEMF: Pulsed electromagnetic field
PGC-1α: Peroxisome proliferator-activated receptor-gamma coactivator-1α
QALYS: Quality-adjusted life-years
RF: Rectus femoris
RFID: Radio frequency identification
RTP: Return-to-play
SF36: Short form 36
SLH: Single leg hop
SPARC: Secreted protein acidic and rich in cysteine
TAU: Treatment-as-usual
TNNT1: C-terminal of troponin T1
TRPC-1: Transient receptor potential canonical 1
VAS: Visual analogue scale
VM: Vastus medialis
VL: Vastus lateralis
