# Supplementary material for: Effect of pulsed electromagnetic field as an intervention for patients with quadriceps weakness after anterior cruciate ligament reconstruction: a double-blinded, randomized-controlled trial
Source: Trials. 2022 Sep 12;23:771. doi: 10.1186/s13063-022-06674-2 (PMC9465849; doi:10.1186/s13063-022-06674-2)
Supplement: Supplementary file 1 — Additional file 1. Informed consent material. [file 13063_2022_6674_MOESM1_ESM.pdf]

Department of Orthopaedics & Traumatology
The Chinese University of Hong Kong
香港中文大學 矯形外科及創傷學系

Patient Information Sheet

''A Double-Blinded, Randomized-Controlled-Trial to Investigate the Effect of Pulsed Electromagnetic Field (PEMF) for patients with quadriceps weakness after Anterior Cruciate Ligament Reconstruction''

Introduction

You are invited to participate in a research study conducted by the Department of Orthopaedics and Traumatology at the Chinese University of Hong Kong. People with anterior cruciate ligament (ACL) injury often suffer knee instability, results in decreased activity level. Despite the success in reconstruction and a demanding rehabilitation process, some athletes still suffer dynamic knee instability and even get a second ACL injury. One of the main reasons for high re-injury rate is the failure to regain the pre-injury muscular function.

Description of the study

Previous research shows that quadriceps muscle strength and knee function of patient in post-ACLR period are related to the recovery. To investigate the muscle atrophic mechanism after ACLR, we would like to study the effectiveness of pulsed electromagnetic field (PEMF) therapy on quadriceps muscle strength for post-ACLR patients. As PEMF treatment may affect the metabolism and muscle growth, we hope to examine the outcome of PEMF treatment on muscle regeneration, serum myokine, and knee function in post-ACLR period.

Procedure

Tegner activity score and muscle strength test will be used for the enrolment screening. Afterwards, you will be randomly assigned to receive either a PEMF treatment or a sham treatment. You will be invited to attend 10-minute intervention sessions twice a week, for 8 weeks, fulfilling a total of 16 sessions. If you cannot attend the scheduled session, please notify in advance, and reschedule for a make-up session. The rescheduled session should be within the week of the missed session. The whole intervention programme has to be completed within 8 weeks.

Before the intervention, you will be asked to complete blood samples drawing, questionnaires, muscle strength test, ultrasound imaging for quadriceps muscles, knee laxity, biomechanical analysis, and bone density and structure scan. At 4- and 8- weeks after the commencement on PEMF treatment, as well as 12 months after ACLR, you will be asked to complete the same set of assessments. MRI scan will be performed before and 8- weeks after the start of PEMF treatment if possible. The whole intervention and assessments will not affect your existing medical care and rehabilitation.

Risks and Benefits

All the treatment and assessments will be completed under the supervision of professional staff in CUHK and will be conducted according to standard procedures. Medical staff in PWH will draw blood samples for patients. In addition, in any case of unexpected or undesirable events, we will follow you closely and keep track of any pain or discomfort after testing. If you experience any pain or discomfort (including swelling, heat, and/or redness) during the testing, you are always allowed to stop the test immediately.

Participation

Your participation is voluntary without any cost and no incentive will be provided for the participation; this means you can choose to withdraw at any time without giving any reason, without your medical care or legal rights being affected. You will only participate in one PEMF related study at a time. If you decide to take part in this research, please fill out and sign the consent form. Researcher will arrange assessment session for you. Please kindly arrive on time for your scheduled appointment. If you would like to reschedule the appointment, please contact the researcher as soon as possible.

Information Protection

Your personal information and data will only be accessed by the principal investigator, researchers involved in the study, and the regulatory authorities. If the results of the study are published, your identity will remain confidential. The researcher will keep the information and samples collected for at least 5 years beyond the end of the study.

Contact person

If you have any inquiries about this study, please contact the principal investigator Prof. Michael Ong at 26364171. You can also acquire your rights via The Joint Chinese University of Hong Kong – New Territories East Cluster Clinical Research Ethics Committee at 35053935.

Department of Orthopaedics & Traumatology
The Chinese University of Hong Kong
香港中文大學 矯形外科及創傷學系

Patient Information Sheet for a research project "A Double-Blinded, Randomized-Controlled-Trial to Investigate the Effect of Pulsed Electromagnetic Field (PEMF) for patients with quadriceps weakness after Anterior Cruciate Ligament Reconstruction''

I,__________________________________,HKID ___________________________ consent to participate in the research project "A Double-Blinded, Randomized-Controlled-Trial to Investigate the Effect of Pulsed Electromagnetic Field (PEMF) for patients with quadriceps weakness after Anterior Cruciate Ligament Reconstruction''

I have read the consent form, understand that the procedure and risks involved and have received answers to any questions I asked. I understood the nature of this study and agree that the information collected will be kept by the researcher for at least **5 years** beyond the end of the study and blood samples collected will be kept in an established tissue bank at Prince of Wales Hospital. I understand that the data collected will be published to the public and in peer-reviewed scientific papers anonymously.

Signature: ___________________

Name: ____________________

Date: ____________________

Signature of person obtaining consent: ____________________

Name of person obtaining consent: ________________________

Date: ______________________

*If you have any inquiries, you can contact the following persons for more information:

Principal investigator:	Professor Ong Michael Tim Yun	Tel: 35052083
Co-investigator:	Dr. Fu Sai Chuen	Tel: 35053311
Joint CUHK-NTEC CREC		Tel: 35053935
