# Supplementary material for: Effect of pulsed electromagnetic field as an intervention for patients with quadriceps weakness after anterior cruciate ligament reconstruction: a double-blinded, randomized-controlled trial
Source: Trials. 2022 Sep 12;23:771. doi: 10.1186/s13063-022-06674-2 (PMC9465849; doi:10.1186/s13063-022-06674-2)
Supplement: Supplementary file 2 — Additional file 2. Institutional ethical approval. [file 13063_2022_6674_MOESM2_ESM.pdf]

Joint Chinese University of Hong Kong-New Territories East Cluster Clinical Research Ethics Committee
香港中文大學-新界東醫院聯網 臨床研究倫理 聯席委員會

8/F, Lui Che Woo Clinical Sciences Building, Prince of Wales Hospital, Shatin, HK
Tel : (852) 3505 3935 / 2144 5926 Fax : (852) 2646 6653 Website : http://www.crec.cuhk.edu.hk

The Joint CUHK-NTEC CREC is an independent committee established by CUHK/NTEC and authorized to perform ethics and scientific review and oversight of clinical studies within the jurisdiction of CUHK/NTEC in accordance with its standard operating procedure and the principles of the Declaration of Helsinki and ICH Good Clinical Practice.

CREC Ref. No.: 2021.332-T

2 SEP '21

To: Prof. Tim Yun ONG
Dept. of Orthopaedics & Traumatology
Prince of Wales Hospital

This notice is issued by the Joint CUHK-NTEC CREC with respect to the application/submission by you, being the principal investigator of the following study at your study site:

- **Study Protocol Title:** A Double-Blinded, Randomized-Controlled-Trial to Investigate the Effect of Pulsed Electromagnetic Field (PEMF) for patients with quadriceps weakness after Anterior Cruciate Ligament Reconstruction
- **Investigator(s):** Tim Yun ONG and Patrick Shu Hang YUNG

In accordance with our standard operating procedure, we have duly performed ethics and scientific review of your application/submission as detailed below:

- **Nature of Your Application/Submission:** ☒ Initial application ☐ Amendments/changes ☐ Others: ☐ Renewal
- **Mode of Review:** ☒ Full review ☐ Expedited review
- **Date of Initial/Renewal Approval:** 30 August 2021
- **Document(s) Reviewed:** See Schedule 1
- **Reviewer(s):** See Schedule 2

After due review by our reviewer(s), we hereby write to inform you of our decision on your application/submission as follows:

- **Decision:**
  - ☐ Application/Submission approved
  - ☒ Application/Submission approved with condition(s) (see condition(s) below)
  - ☐ Application/Submission approved with remark(s) (see remark(s) below)
  - ☐ Application/Submission approved with condition(s) and remark(s) (see condition(s) and remark(s) below)
- **Condition(s):** A copy of the Certificate for Insurance is required to be submitted to the Joint CUHK-NTEC CREC prior to commencement of the study

Joint Chinese University of Hong Kong-New Territories East Cluster
Clinical Research Ethics Committee
香港中文大學-新界東醫院聯網 臨床研究倫理 聯席委員會

8/F, Lui Che Woo Clinical Sciences Building, Prince of Wales Hospital, Shatin, HK
Tel : (852) 3505 3935 / 2144 5926 Fax : (852) 2646 6653 Website : http://www.crec.cuhk.edu.hk

2 SEP '21

- **Regular Progress Report(s) Required:** Every 12 months from the date of initial/renewal approval and during the period of the study if required

You, being the principal investigator of the study at your study site, are reminded to comply with our requirements and to maintain communication with us during the period of the study by undertaking the principal investigator's responsibilities including (but not limited to):

- if the study is an industry-sponsored clinical study, submitting to us a copy of the fully executed indemnity agreement satisfying the Hospital Authority's requirement prior to commencement of the study (if it has not been submitted yet);
- observing and complying with all applicable requirements under our standard operating procedure ("IRB/REC SOP"), the Declaration of Helsinki and the ICH GCP (if applicable);
- submitting regular progress report(s) at the required intervals (as specified above) in accordance with the requirements in the IRB/REC SOP;
- not implementing any amendment/change to any approved study document/material without our written approval, except where necessary to eliminate any immediate hazard to the subjects or if an amendment/change is only of an administrative or logistical nature;
- notifying us of any new information that may adversely affect the rights, safety or well-being of the subjects or the proper conduct of the study;
- reporting any deviation from the study protocol or compliance incident that has occurred during the study and may adversely affect the rights, safety or well-being of any subject in accordance with the requirements in the IRB/REC SOP;
- submitting safety reports on all SAEs observed at your study site or SUSARs reported from outside your study site in accordance with the requirements in the IRB/REC SOP; and
- submitting a final report in accordance with the requirements in the IRB/REC SOP upon completion or termination of the study at your study site.

In addition to the above, you are also reminded to observe and comply with other applicable regulatory and management requirements including (but not limited to):

- if required by Hong Kong laws or regulations, obtaining a certificate for clinical trial through the Hong Kong Department of Health and complying with the associated requirements;
- obtaining the necessary consent from the management of your institution/department in accordance with the requirements of your institution/department;
- if required by local laws or regulations at conducting site out of IRB/REC's jurisdiction, obtaining an approval and complying with associated requirements;
- not representing to any third party or in any way likely to mislead any third party forming the view that the approval from the IRB/REC has any extraterritorial effect; and
- with due diligence ensuring your teams, staff, agents or whosoever connected with you to comply with the preceding requirements.

Yours sincerely,

Envy Lee (Secretary)
for and on behalf of
The Joint CUHK-NTEC CREC

EL/ci

Schedule 1
Documents Reviewed

The documents reviewed by with respect to the said application/submission include:

- Research Protocol (PEMF_Post-ACLR_Research_Protocol_v01_11MAY2021)
- Patient Information Sheet and Consent Form, English Version
  (PEMF Post ACLR Eng ver v1 23JUL2021) - clean and track changes version
- Patient Information Sheet and Consent Form, Chinese Version
  (PEMF_Post-ACLR_Chi_v01_23JUL2021) - clean and track changes version
- Appendix 1 Lysholm Knee Scoring System, Chinese Version
  (Appendix_1_Lysholm_Chi_v01_09MAY2021)
- Appendix 2 Lysholm Knee Scoring System, English Version
  (Appendix_2_Lysholm_Eng_v01_09MAY2021)
- Appendix 3 International Knee Documentation Committee (IKDC), Chinese Version
  (Appendix_3_IKDC_Chi_v01_09MAY2021)
- Appendix 4 International Knee Documentation Committee (IKDC), English Version
  (Appendix_4_IKDC_Eng_v01_09MAY2021)
- Appendix 5 Tegner Activity Score, Chinese Version
  (Appendix_5_Tegner_Chi_v01_09MAY2021)
- Appendix 6 Tegner Activity Score, English Version
  (Appendix_6_Tegner_Eng_v01_09MAY2021)
- Appendix 7 International Physical Activity Questionnaire (IPAQ), Chinese Version
  (Appendix_7_IPAQ_Chi_v01_09MAY2021)
- Appendix 8 International Physical Activity Questionnaire (IPAQ), English Version
  (Appendix_8_IPAQ_Eng_v01_09MAY2021)
- Appendix 9 The Numeric Pain Rating Scale Instructions, English Version
  (Appendix_9_VAS_Eng_v01_09MAY2021)
- Appendix 10 Project Timeline, English Version
  (Appendix_10_Project timeline_Eng_v01_09MAY2021)

Schedule 2
Reviewers List – Group 2
Joint CUHK-NTEC Clinical Research Ethics Committee

Title and Name	Occupation	Qualification	Male / Female (M/F)	Study Reviewed by	Present in CREC meeting on 01 Jun 2021
**Vice/Deputy Chairman:** Dr. Gary C.P. CHAN	Associate Consultant Department of Medicine and Therapeutics, PWH	MBChB, MRCP (UK), FHKCP, FHKAM	M	√	√
Prof. Peter K.F. CHIU	Associate Professor, Department of Surgery, CUHK	MBChB (CUHK), FRCSEd (Urol), FCSHK, FHKAM (Surg)	M	√	√
Prof. Alexander Yuk Lun LAU	Assistant Professor, Dept. of Medicine and Therapeutics, CUHK	SB, MBChB, MRCP, FHKCP, FHKAM (Medicine)	M	√	
Prof. Liona C.Y. POON	Professor (Clinical), Department of Obstetrics and Gynaecology, CUHK	MBBS (Lond), MRCOG MD(Res) (Lond), Cert RCOG (Maternal and Fetal Med), MSc in Medical Genetics (CUHK)	F	√	√
Prof. Eliza L.Y. WONG	"Professor, JC School of Public Health & Primary Care, Centre for Health Systems and Policy Research, CUHK"	BSN (Canada), MPH (CUHK), PhD (CUHK), PgD Policy Studies (Loud), RN (Canada), RN (HK), FHKCHSE (HK), FCHSE (Australia), Accredited Mediator (HKMAAL, HKIAC), Accredited PASS Supervisor (Australia)	F	√	
Dr. Brian Kai Ming AU	Senior Occupational Therapist, Occupational Therapy Department, TPH	PDOT(HKP), MSc(HKPU), PhD(HKPU)	M	√	
Dr. Albert Kam Ming CHAN	Associate Consultant, Department of Amaesthesia & Intensive Care, PWH	MBBS, FHKCA, FHKAM, FANZCA	M		
Dr. Bosco H.M. MA	Consultant, Department of Medicine & Geriatrics, SH	MBChB (CUHK), MD (CUHK), FRCP (Lond, Edin & Glasg), FHKCP, FHKAM (Medicine)	M		
Dr. Oscar W.H. WONG	Assistant Professor, Department of Psychiatry, CUHK	MBChB (CUHK); FHKAM(Psychiatry); FHKCPsych	M	√	

Title and Name	Occupation	Qualification	Male / Female (M/F)	Study Reviewed by	Present in CREC meeting on 01 Jun 2021
Dr. Keary R. ZHOU	Lecturer, School of Pharmacy, CUHK	BS(UCLA), PharmD(USC)	F	√	√
Ms. Suzanne So Shan MAK	Nurse from Dept. of Clinical Oncology, PWH	RN, MN, FHKAN (Medicine-Oncology)	F		√
Ms. Sylvia Po Yi CHENG	Associate, Morrison & Foerster LLP	PCLL (HKU)<br>BA Law and Business Studies (Warwick)	F		
Ms. Kristy K.Y. CHEUNG	CEO, The Hong Kong College of Anaestheiologists	BSc, MAEP	F	√	
Mr. Christopher K.S. LIU	Executive Director, Liu Chong Hing Investment Ltd.	Bachelor of Arts (Oxford), Master of Arts in Jurisprudence (Oxford)	M	√	
Mr. Ping Hei TAO	Retired	MHRM(MQU), Dip Soc Sci(HKBU)	M	√	√
Ms. Olivia T.L. TO	Registered Nurse	BSc (Nursing), MSc in Cardiology	F	√	√
Mr. Foster H.C. YIM	Barrister-at-Law	PCLL(CUHK), D(CUHK), Msc in Marketing (CUHK), MA in Philosophy (UK), BA (Hons) in Translation (LU)	M		
