# Supplementary material for: Effect of pulsed electromagnetic field as an intervention for patients with quadriceps weakness after anterior cruciate ligament reconstruction: a double-blinded, randomized-controlled trial
Source: Trials. 2022 Sep 12;23:771. doi: 10.1186/s13063-022-06674-2 (PMC9465849; doi:10.1186/s13063-022-06674-2)
Supplement: Supplementary file 4 — Additional file 4. Research protocol (dated 11 May 2021). [file 13063_2022_6674_MOESM4_ESM.docx]

**Research Protocol**

**Title:**A Double-Blinded, Randomized-Controlled-Trial to Investigate the Effect of Pulsed Electromagnetic Field (PEMF) for Patients with quadriceps muscle weakness after Anterior Cruciate Ligament Reconstruction

**Background**

In Hong Kong, over 3000 Anterior Cruciate Ligament Reconstructions (ACLR) are performed each year in order to restore knee function after an ACL injury. The ultimate goal of ACLR is to fulfil the return-to-play (RTP) criteria. Despite successful surgery and a demanding rehabilitation process, some athletes still fail to comply to RTP. And for those who achieve RTP, 23% of those who return to their sports would suffer a second ACL injury (1).

After ACL injury, the patients would undergo ACLR and post-operative rehabilitation. The rehabilitation gradually helps patients to regain mobility and strength, where most of the patients would be expected to RTP by 12 months. Despite comprehensive rehabilitation programs, a systematic review showed up to 35% of patients would fail to return to their preinjury level of play (2). Additionally, these patients were found to be 6 times more likely to sustain a second ACL injury within 24 months after ACLR than the healthy population (3).

Quadriceps muscle strength is one of the key determinants for a patient’s successful return-to-play after ACLR, by which muscle size is a crucial factor of muscle strength. Quadriceps muscle atrophy is unavoidable after ACLR, while rehabilitation aids in recovering quadriceps muscle mass. However, despite good compliance, some patients respond poorly and fail to regain muscle mass. Quadriceps muscle atrophy can persist beyond the completion of the rehabilitation program in almost half the patients and the reason behind this is still unknown. This represents an area requiring significant investigation, as quadriceps muscle atrophy and weakness have been shown to be predictive of poor knee function, decreased performance in sports and increased risk of reinjury.

Graft-tunnel osteointegration is another key determinant for the successful outcome of ACLR. Tunnel widening and bone loss are common after ACLR which affect tendon graft fixation and subsequent osteointegration of the tendon graft. Transient loss of 5–10% of bone mineral density (BMD) around the knee has been found in our previous clinical study (19). The tunnel surface with decreased BMD can become less stable for tendon–bone integration due to increased bone resorption. A successful osteointegration of the tendon graft with normal BMD is vital for the rehabilitation of ACLR, as early stability of knee joint allows the patients to adopt more aggressive rehabilitation therapy and improve the outcomes of surgery. In addition, patients having a higher degree of knee pain were found to have a higher degree of bony sclerosis and overall subchondral osteopenia and were associated with more quadriceps atrophy. Thus, the bone microarchitectural evolution after ACLR is worth monitoring.

PEMF treatment can modulate mitochondrial activities for muscle gain. Molecular processes are sensitive to the magnetic flux at extremely low frequency (<300 Hz), which are equivalent to the Earth’s natural magnetic resonance (Schumann resonance). Applying magnetic fields at these low frequencies in the form of magnetic pulses, like a timely push to swing. The pulsing action will enhance the human magnetosensitive processes, which include the electron transport chain in mitochondria, calmodulin signaling and ion channels. The affected signal transduction pathways further influence the downstream myogenesis (5). Therefore, a pulsed electromagnetic field (PEMF) may be a possible intervention to amplify molecular processes in favour of myogenesis (6).

PEMF therapy has been demonstrated to enhance cellular activity related to tissue healing (7), and offer beneficial effects such as pain relief, anti-inflammation and oedema resolution (8). Numerous variables in the delivery of PEMF signals (i.e., magnetic flux, frequencies, signal curve, polarity, etc) may have specific biological implications. Our group (Prof. Alfredo Franco-Obregon) showed that brief 10-minute exposure of 1.5mT amplitude of PEMF (Quantum Tx) on myoblast in-vitro could activate myogenesis. It is done so by stimulating the TRPC-1-mediated calcium entry and downstream factors as well as PGC-1α, this mechanism (mentioned above) is similar to the myogenesis in exercise (4). PGC-1α gene expression is influenced by every major signaling pathway that is activated in a contracting muscle (9). Additionally, it plays an important role in regulating the production and secretion of myokine. Therefore, PEMF exposure on top of regular exercise training, may promote the secretion of myokine and in turn promote muscle regeneration.

Our study will examine the efficacy of PEMF treatment introduced at the late postoperative period. It helps to set up the treatment regime for the PEMF device to impose myogenic effects, including exposure time per session, the number of treatment sessions and the duration of treatment. To our best knowledge, this proposed study aims to apply PEMF as an intervention for patients with myokine-mediated persistent quadriceps muscle atrophy after ACLR. In turn, it would be the first study to examine the correlation between PEMF and post-operative quadriceps muscle endurance, strength and mass. Apart from the quadriceps muscle, we will also examine the bone microarchitecture changes correlating to the PEMF treatment in the post-operative period.

**Objective**

To conduct a double-blinded, randomized controlled trial to investigate the effects of PEMF treatment during the late postoperative period on quadriceps muscle strength and muscle weakness in ACL injured patients.

**Hypothesis**

PEMF treatment is effective to reduce muscle weakness and promote a gain in quadriceps muscle strength for ACLR patients at the post-operation time points.

**Setting**

The study will be carried out in the Department of Orthopaedics and Traumatology at Prince of Wales Hospital.

**Subject recruitment**

The study protocol will comply with the Declaration of Helsinki and ICH-GCP. Subjects will be recruited from the Department of Orthopaedics and Traumatology of Prince Wales Hospital in Hong Kong and patients who can meet all the inclusion criteria will be invited to participate in two sessions of assessments.

A total of 40 post-ACLR subjects will be recruited and follow up for up to 12 months. They will undergo 1:1 randomization into two study arms:

1. PEMF treatment
2. Control (Sham treatment)

Inclusion criteria:

1. Aged 18-50 with a unilateral ACL injury
2. Sporting injury with a Tegner score of 7+
3. LSI for quadriceps strength <70% of the contralateral leg at 4 months post-op
4. Both knees without a history of injury/prior surgery

Exclusion criteria:

1. Ages smaller than 18 years old or greater than 50 years old
2. Any concomitant bone fracture, major meniscus injury or full-thickness chondral injuries requiring altered rehabilitation program post-op
3. Preoperative radiographic signs of arthritis
4. Metal implants that would cause interference on MRI
5. Non-HS graft for ACLR
6. Patient non-compliance to the rehabilitation program
7. Pregnancy or possibility of pregnancy

**Method**

Intervention

The intervention will be held at Prince of Wales Hospital twice a week. Participants in the intervention group will be exposed to PEMF treatment by a PEMF device (Quantum Tx, Singapore). The active pulse electromagnetic field device does not produce heat or cause any sensation to the tissue which allows the participants to be blinded to the treatment. Participants in the control group will receive a sham exposure with the same PEMF device. The involved leg will be exposed to PEMF or sham treatment for 10 minutes per session, and the treatment regime will run biweekly for 8 weeks, summing up 16 sessions of PEMF or sham exposure in total. According to our pilot study in Singapore, such an arrangement establishes a cross-feeding scenario whereby muscle recovery is fully supported and systemic myokine release is optimized. The procedure of PEMF treatment is shown as follows:

- The subject will be seated at a 90 degrees position on a chair.
- The solenoids of the PEMF device will be adjusted to be over the thigh (quadriceps and hamstring).
- The options of the appliance will be adjusted to 1 mT, 15Hz on one leg for 10 minutes.

**Outcome measures**

Isokinetic muscle strength

The dynamometer (Biodex System 4, Biodex Medical Systems Inc., New York, USA) will be used. Subjects will perform a standardized warm-up exercise (5 min cycling) followed by the fatigue test. 5 repetitions at speed of 180°/s will be done first to familiarize the patient. Concentric/concentric contractions of knee extension/flexion will be tested at 180°/s for 30 repetitions. Subjects will be seated on the dynamometer chair with the hip flexed to 85°. The re-test reliability has been proven for this protocol (11). The relatively fast speed is chosen to perform the fatigue test since the fast-twitch muscle fibres are expected to fatigue faster at high speeds (12). Peak torque will be the single highest repetition value within the 30 repetitions (13). Muscle endurance could be reflected by fatigue test, where the trends for peak torque, work, and power will be examined. Fatigue Index (F.I.) will be used to calculate the percent decrease for each variable (13). Percent decrease = 100 – [(last 5 repetitions/ first 5 repetitions) x 100]. For individuals that did not achieve their peak torque within the first 3 repetitions, a second F.T. will be calculated as follows. Percent decrease = [100 – [(last 5 repetitions/ highest consecutive 5 repetitions) x 100], where the highest consecutive five repetitions will be determined by values attained from the repetitions immediately before and following, the single highest repetition value (13).

Self-reported knee functions

The subjective functional outcome will be evaluated by the Lysholm knee scoring system, the International Knee Documentation Committee (IKDC) subjective scoring system, Tegner activity score and International Physical Activity Questionnaire (IPAQ) will be used.

1) Lysholm knee scoring system: Consists of eight items, total score ranging from 0 to 100 where higher scores indicate a better outcome with fewer symptoms or disability (14).

2) International Knee Documentation Committee (IKDC): Consisted of 10 questions on symptoms and activity ranging from 0 to 100 where 100 implies perfect knee function (15).

3) Tegner activity score: This is an activity level scaled from 1 (low activity) to 10 (high activity) (16).

4) International Physical Activity Questionnaire (IPAQ): The level of physical activities during the past 7 days will be evaluated with a validated Chinese version of the quantitative physical activity questionnaire (17).

Muscle thickness

1. Ultrasound Imaging

The Aixplorer® ultrasound system (SuperSonic Imagine, Aix-en-Provence, France) and a linear transducer probe with a bandwidth of 2-10 MHz (SuperLinear™ SL10-2, Vermon, Tours, France) were used to measure the muscle thickness of Vastus Medialis (VM), Vastus Lateralis (VL), and Rectus Femoris (RF) on both the injured and uninjured leg. Participants laid supine on a treatment table for the assessment. A measuring tape was used to locate VM, VL, RF and the patella by palpation, consequently marked with a pen for reference. For consistency and ease of comparison across patients, the locations measured and labelled as the three muscle groups follow the guidelines below. RF was marked at 1/2 of the distance from the anterior superior iliac spine (ASIS) to the superior pole of the patella, VM was located at 1/5 of the distance away from the midpoint of the medial patella border to the ASIS, and VL was noted at 1/3 of the distance from the midpoint of the lateral patella border to the ASIS. After locating the anatomical points, excess contact gel was applied on these points. The transducer probe was aligned in the transverse plane and moved along the entire muscle bundle to capture a view of the VM, VL and RF. The operator would position the probe into the sagittal plane to measure muscle thickness upon the marked anatomical points. Minimal pressure was applied on the limb to prevent the deformation of the muscle and the results would be derived from the average of three measurements.

1. Magnetic Resonance Imaging (MRI)

Muscle volumes of quadriceps muscle are measured using a 1.5 or 3.0 Tesla MRI Scanner. Axial (3mm thick cut) T1W images are obtained from the anterior superior iliac spine (ASIS) to the patella. Quadriceps muscles were manually outlined in each axial slice. Muscle volume was calculated by summing all of the slice-multiplied by slice thickness. The quality of the muscle is assessed by analyzing the fat content of the muscle mass using a technique that has been reported by Reeder et al (15). Scans will be performed on both legs before the start of the PEMF treatment (4 months post-op) and only on the injured side repeated after the completion of the 8 weeks PEMF treatment (6 months post-op). The uninjured side will be used as a reference for ‘normal volume’.

High-Resolution Peripheral Quantitative Computed Tomography Scans (HR-pQCT)

High-resolution peripheral quantitative computed tomography scans (HR-pQCT) using XtremeCT II (Scanco Medical AG®, Brüttisellen, Switzerland) provides an analysis of bone microarchitecture at human peripheral sites with high spatial resolution and low exposure to radiation. Bone mineral density (BMD, calibrated with hydroxyapatite standards) and finite element analysis of subchondral bone over the knee joint will be performed using the Scanco software. Scans will be performed on both legs before the start of the PEMF treatment (4 months post-op), and on the injured side after the completion of the PEMF treatment (6- and 12-months post-op). The uninjured side will be used as a reference for ‘normal’ bone condition.

Biomechanical analysis

The kinematics will be assessed by the skin marker-based motion analysis system (Vicon MX, Oxford, UK) with the lower-body marker setup followed the OSTRC standard protocol using 16-camera and 16 reflective skin markers. The kinetic variables including vertical and horizontal ground reaction force (GRF) and joint moments will be evaluated by a synchronized force plate (0.60 × 0.40 m, model OR6-7, AMTI, Watertown, Massachusetts) at the centre of the capture volume at 1000 Hz. From the SLH task, the knee kinematics in the landing phase is the key measurements including knee flexion angle at initial contact and the range of knee motions (flexion-extension, valgus-varus, external-internal rotation). The hip and ankle flexion angle at initial contact will also be recorded. The GRF will be normalized to body weight and moments will be normalized for body weight ×limb length to make the comparison between subjects possible. All analyses for moments and angles were performed in the anatomical sagittal (XY) plane and are counted as positive for flexion angles and extension moments. The start of the rise in the vertical GRF will be used to determine initial ground contact. From the SLS task, the knee kinematics are the key measurements including the range of motions (flexion-extension, valgus-varus, external-internal rotation) and the frequency of the relative tibiofemoral angular displacements from the kinematics curve, which will be quantified by the number of peak appearance. The kinematic and kinetic variables were imported and calculated in the Matlab (The Mathworks Inc., Natick, Massachusetts, USA). A biomechanical analysis will be performed on all patients. At the same time, the motion will be videotaped by high-speed cameras.

Serum myokine evaluation

Phlebotomy (5ml) will be performed on the day before PEMF treatment, at 4- and 8- weeks after commencement of treatment, and at post-op 12 months. The serum will be prepared by centrifugation and kept in a -80o freezer until use. Quantitative analysis for myokines and proteins related to muscle metabolism will be performed by Human Myokine Magnetic Bead Panel (Millipore) with Bioplex-200 bead-based suspension assay system (LKSIHS core facilities), or enzyme-linked immunosorbent assay (ELISA). These include Brain-derived neurotrophic factor (BDNF), Fibroblast growth factor-21 (FGF-21), Interleukin-6 (IL-6), IL-15, Irisin, Myostatin (MSTN)/GDF8, Insulin-like growth factor 1 (IGF-1), FGF-2, IL-8, Follistatin, Musclin, Myonectin, Decorin, Meteorinlike, Osteopontin, Secreted protein acidic and rich in cysteine (SPARC), Klotho, Procollagen type III N-terminal peptide (P3NP), and C- terminal of troponin T1 (TNNT1).

Passive Knee laxity

To measure anterior-posterior knee laxity, the KT-1000 knee ligament arthrometer (MEDmetric Corp, San Diego, CA, USA) will be used. A manual force test will be applied until a 30lb sound signal is activated. Three trials will be performed. A side difference of 3 mm above is considered clinically relevant.

Visual Analogue Scale

The subjective measurement for chronic and acute pain will be recorded by the Visual Analogue Scale (VAS). VAS consists of a 10-cm line which represents the continuum between “painless” and “worst pain from 0 cm to 10 cm. The subject will be asked to draw a mark of it before and after each PEMF treatment session.

**Study design**

Randomization and blinding

Participants will be randomized into 1:1 allocation. Blocked randomization will be undertaken based on the computer-generated allocation sequence, whereby 40 participants would be divided into blocks of 10 for 2 groups, Thereafter, the participants will use their key card to complete the assigned treatment regime, without knowing if the machine is delivering active or sham PEMF. Outcome assessors and trial administrators are blinded for the group allocation.

Follow up time points

Subjects with a Tenger Score 7+ and with ≥ 7% of the deficit in total quadricep volume on the involved leg compared with the uninvolved leg will be recruited into a randomized controlled trial. Baseline assessments including isokinetic quadriceps muscle strength, quadricep muscle thickness, blood sampling for myokines measurement (5ml), biomechanical analysis, knee laxity and self-reported outcome with a questionnaire will be taken before commencement of treatment, and the same set of measurement will be monitored at 4- and 8- weeks after commencement of PEMF or sham treatment. MRI will be performed before PEMF and 8- weeks after the commencement of PEMF if possible. And at post-op 12 months, isokinetic quadriceps muscle strength, quadricep muscle thickness, biomechanical analysis and questionnaire will be taken again. (Appendix 10)

**Sample size calculation**

Quadriceps muscle endurance and strength will be employed as the primary outcome for sample size estimation. When the sample size is 14 per groups with 3 repeated measurements (baseline, 4- and 8- weeks after commencement of PEMF), a repeated-measures ANOVA will give 95% of power to detect at the 0.05 level an effect size of 0.58 for between-subject factor based on the previous research (18) (G*Power 3.0 software). An additional 40% will be added to account for possible attrition. Thus, 40 (20x2) subjects will be recruited in total.

**Data processing and analysis**

The data obtained in the tests will be analyzed using Statistical Package for Social Science (SPSS) (IBM SPSS ver.26). For parametric data, a paired-sample t-test will be used. Wilcoxon signed-rank test will be used to analyze the data sets if they are non-parametric. The alpha level will be set at 0.05 for all statistical tests.

**Potential pitfall and contingency plan**

Patients that are potentially intolerable for magnetic low-field magnetic stimulation or trigger discomfort will not be included in our study during screening. If the patient record did not indicate any potential adverse reaction to the PEMF treatment and experience such a situation during the intervention, the patient would be immediately removed from the study in their best interest to safeguard their health. All staff operating the PEMF would have thorough training on the operation of the machine and familiarize themselves with the troubleshooting scheme. Study personnel would indicate and explain to patients during recruitment about the intervention duration and frequency to stress the importance of compliance, accompanied with frequent reminders to participants. In the case of patient non-compliance, study personnel would remove the patient from the study only when multiple failed attempts to contact and convince the patient occurs.

References:

1. Wiggins AJ, Grandhi RK, Schneider DK, Stanfield D, Webster KE, Myer GD. Risk of secondary injury in younger athletes after anterior cruciate ligament reconstruction: a systematic review and meta- analysis. The American journal of sports medicine. 2016;44(7):1861-76.

2. Ardern CL, Taylor NF, Feller JA, Webster KE. Fifty-five per cent return to competitive sport following anterior cruciate ligament reconstruction surgery: an updated systematic review and meta- analysis including aspects of physical functioning and contextual factors. British journal of sports medicine. 2014;48(21):1543-52.

3. Paterno MV, Rauh MJ, Schmitt LC, Ford KR, Hewett TE. Incidence of second ACL injuries 2 years after primary ACL reconstruction and return to sport. The American journal of sports medicine. 2014;42(7):1567-73.

4. Yap JLY, Tai YK, Fröhlich J, Fong CHH, Yin JN, Foo ZL, et al. Ambient and supplemental magnetic fields promote myogenesis via a TRPC1-mitochondrial axis: evidence of a magnetic mitohormetic mechanism. The FASEB Journal. 2019;33(11):12853-72.

5. Giusti A, De Vincentiis A, Fratoni F, Giovale M, Bianchi G. Effect of Repeated Application of Low-Intensity Pulsed Electromagnetic Fields (PEMF) on Gait Speed in Older Adults with a History of Falls. Journal of the American Geriatrics Society. 2014;62(6):1185-6.

6. Liu M, Lee C, Laron D, Zhang N, Waldorff EI, Ryaby JT, et al. Role of pulsed electromagnetic fields (PEMF) on tenocytes and myoblasts—potential application for treating rotator cuff tears. Journal of Orthopaedic Research. 2017;35(5):956-64.

7. Ay S, Evcik D. The effects of pulsed electromagnetic fields in the treatment of knee osteoarthritis: a randomized, placebo-controlled trial. Rheumatology international. 2009;29(6):663-6.

8. Abdelhalim NM, Samhan AF, Abdelbasset WK. Short-Term impacts of pulsed electromagnetic field therapy in middle-aged university's employees with non-specific low back pain: A pilot study. Pakistan journal of medical sciences. 2019;35(4):987.

9. Schnyder S, Handschin C. Skeletal muscle as an endocrine organ: PGC-1α, myokines and exercise. Bone. 2015;80:115-25.

10. Konishi Y, Ikeda K, Nishino A, Sunaga M, Aihara Y, Fukubayashi T (2007) Relationship between quadriceps femoris muscle volume and muscle torque after anterior cruciate ligament repair. Scandinavian journal of medicine & science in sports 17:656-661

11. Pincivero D, Lephart S, Karunakara R. Reliability and precision of isokinetic strength and muscular endurance for the quadriceps and hamstrings. International journal of sports medicine. 1997;18(2):113-7.

12. So R, Chan K-M, Siu O. EMG power frequency spectrum shifts during repeated isokinetic knee and arm movements. Research quarterly for exercise and sport. 2002;73(1):98-106.

13. Pincivero DM, Gandaio CB, Ito Y. Gender-specific knee extensor torque, flexor torque, and muscle fatigue responses during maximal effort contractions. European journal of applied physiology. 2003;89(2):134-41.

14.Kocher MS, Steadman JR, Briggs KK, Sterett WI, Hawkins RJ. Reliability, validity, and responsiveness of the Lysholm knee scale for various chondral disorders of the knee. JBJS. 2004;86(6):1139-45.

15. Irrgang JJ, Anderson AF, Boland AL, Harner CD, Kurosaka M, Neyret P, et al. Development and validation of the international knee documentation committee subjective knee form. The American journal of sports medicine. 2001;29(5):600-1

16. Tegner Y, Lysholm J. Rating systems in the evaluation of knee ligament injuries. Clinical orthopaedics and related research. 1985(198):43-9.

17. Baecke JA, Burema J, Frijters JE. A short questionnaire for the measurement of habitual physical activity in epidemiological studies. The American journal of clinical nutrition. 1982;36(5):936- 42.

18. Fu, Chak Lun Allan, et al. The effect of early whole-body vibration therapy on neuromuscular control after anterior cruciate ligament reconstruction: a randomized controlled trial. The American journal of sports medicine 41.4 (2013): 804-814.

19. Lui, P. P., Cheng, Y. Y., Yung, S. H., Hung, A. S., & Chan, K. M, et al. A randomized controlled trial comparing bone mineral density changes of three different ACL reconstruction techniques. The Knee 2012 19(6): 779–785
