# Supplementary material for: Effect of pulsed electromagnetic field as an intervention for patients with quadriceps weakness after anterior cruciate ligament reconstruction: a double-blinded, randomized-controlled trial
Source: Trials. 2022 Sep 12;23:771. doi: 10.1186/s13063-022-06674-2 (PMC9465849; doi:10.1186/s13063-022-06674-2)
Supplement: Supplementary file 3 — Additional file 3. Proof of funding. [file 13063_2022_6674_MOESM3_ESM.pdf]

醫療衞生研究基金
Health and Medical Research Fund

Research Fund Secretariat, Research Office, Food and Health Bureau
9th Floor, Rumsey Street Multi-storey Carpark Building, 2 Rumsey Street, Sheung Wan, Hong Kong
Tel: (852) 3150 8963 Fax: (852) 2102 2444 E-mail:rfs@fhb.gov.hk

RESEARCH COUNCIL

Chairperson

Secretary for Food and Health / Permanent Secretary for Food and Health (Health)
食物及衞生局局長／食物及衞生局常任秘書長(衞生)

Members

Prof Juliana CHAN 陳重娥教授
Dr Vincent CHENG 鄭智聰醫生
Prof Timothy KWOK 郭志鋭教授
Prof Linda LAM 林翠華教授
Prof LAU Chak-sing 劉澤星教授
Prof LEUNG Suet-yi 梁雪兒教授
Prof LIN Chia-chin 林佳靜教授
Prof Vivian LIN 林光汶教授
Prof LYU Aiping 呂愛平教授
Prof David MAN 文偉光教授
Prof Tony MOK 莫樹錦教授
Prof Hextan NGAN 顏婉嫦教授
Prof David SHUM 岑浩強教授
Prof Samuel WONG 黃仰山教授
Prof YIP Shea-ping 葉社平教授
Secretary for Innovation and Technology or representative 創新及科技局局長或代表
Director of Health or representative 衞生署署長或代表
Chief Executive of Hospital Authority or representative 醫院管理局行政總裁或代表
Dean of the Faculty of Medicine of The Chinese University of Hong Kong or representative 香港中文大學醫學院院長或代表
Dean of the Li Ka Shing Faculty of Medicine of The University of Hong Kong or representative 香港大學李嘉誠醫學院院長或代表

Our Ref.: FHB/H/41/187
Your Ref.:

Tel: 3150 8920
Fax: 2102 2444

8 April 2022

Prof Michael ONG Tim-yun
Clinical Assistant Professor
Department of Orthopaedics and Traumatology
The Chinese University of Hong Kong
Room 74049, 5/F, Lui Che Woo Clinical Sciences Building
30-32 Ngan Shing Street, Shatin, New Territories, Hong Kong

Dear Prof Ong,

Health and Medical Research Fund
Grant Application (Ref: 19202461)

I am pleased to inform you that the Research Council has approved a grant of HK$1,405,920 for your application titled *"A double-blinded, randomized-controlled-trial to investigate the effects of pulsed electromagnetic field (PEMF) for patients with quadriceps muscle weakness after anterior cruciate ligament reconstruction (ACLR)"*. The Agreement has been sent to the research office of your institution. They will liaise with you for signing the Agreement.

I would like to highlight the following for your special attention -

(a) Please complete the enclosed declaration form.

(b) The commencement date of this project should be within 6 months of the date of this letter.

(c) You and your witness are required to sign three copies of the Agreement.

(d) The terms and conditions on reporting requirements, ethics/ regulatory approval, intellectual property rights and administrative issues are summarised at **Annex**.

(e) The copy of the Application with modifications (if any) accepted by the Government, is accessible in the electronic Grant Management System (eGMS) (https://rfs.fhb.gov.hk/eGMS/).

(f) Please return the signed copies of the Agreement together with the declaration form to the Research Fund Secretariat **via the research office of your institution by 6 May 2022.**

Declaration: Approved project funded by
Health and Medical Research Fund (HMRF)

Please return together with signed agreements to the Secretariat by **6 May 2022**.

Project Reference: 19202461

Project Title: A double-blinded, randomized-controlled-trial to investigate the effects of pulsed electromagnetic field (PEMF) for patients with quadriceps muscle weakness after anterior cruciate ligament reconstruction (ACLR)

Principal Applicant: Prof Michael ONG Tim-yun

Institution: The Chinese University of Hong Kong

I hereby declare that

1. The captioned project has not received and will not receive funding from other sources;

2. # No research work under the captioned project was conducted before the Commencement Date stated in the Agreement.

3. * (a) Regulatory/ ethics approvals have been obtained for the captioned project. The protocol/ scope approved by the relevant regulatory body/ Institutional Review Board is the same as that approved by HMRF; **or**

   * ~~(b)~~ Regulatory/ ethics approvals are not required for the captioned project.

[signature]

(Principal Applicant's signature)

11/April/2022

(Date)

# If not, please provide details (including the budget under the captioned project for the relevant work) and explanation in a separate sheet for Government's consideration.

* delete where appropriate

Summary of the terms and conditions

(a) **Clause 4** on the reporting requirements. Please note the types of reports to be submitted and deadline for each submission. The Dissemination Report may, upon acceptance by the Grant Review Board, be published by the Hong Kong SAR Government and indexed online for wider dissemination to the research community.

(b) **Clause 5** on payment arrangement. Claims for reimbursement shall only cover the period between Commencement Date and End Date (both dates inclusive) stipulated in the **Third Schedule**.

(c) **Clause 10** on intellectual property rights. The Principal Applicant and the Administering Institution shall not use the materials for any purpose other than non-commercial academic purpose. Written consent from the Government is required to assign intellectual property rights (including copyright) to a third party.

(d) **Clause 11** on Invention. You are reminded that the Hong Kong SAR Government co-owns with the Administering Institution all materials and deliverables (including copyright) resulting from this project.

(e) **Clause 15** on procurement of equipment, goods and services.

(f) **Clause 16** on recruitment of project staff.

(g) **Clause 33** on reporting conflict of interest. The Principal Applicant and the Institution shall notify the Government in writing of all or any facts which may reasonably be considered to give rise to a situation where the financial, professional, commercial, personal or other interests of the Principal Applicant or the Institution or any of their respective associates and associated persons, or any member of the Project Team (i.e. Co-applicant(s), conflict or compete, or may conflict or compete, with the Principal Applicant's or the Institution's duties to the Government under this Agreement.

(h) **Clause 34** on the no double funding requirement. The Project Team shall not submit the same or similar project proposal to, or receive financial assistance for the same or similar project proposal from, any other funding agency or this Fund.

(i) **Second Schedule** on clearance requirements.

(j) **Fifth Schedule** for governance of incentives and payments.

(k) Annex for Special Condition:
The Principal Applicant shall report to the Government on the progress of study participants recruitment 6 months after project commencement.
